# Structure–function relationships for squid skin-inspired wearable thermoregulatory materials

**DOI:** 10.1063/5.0149289

**Published:** 2023-11-06

**Authors:** Panyiming Liu, Erica M. Leung, Mohsin Ali Badshah, Christopher S. Moore, Alon A. Gorodetsky

**Affiliations:** 1 Department of Materials Science and Engineering, University of California, Irvine, California 92697, USA; 2 Department of Chemical and Biomolecular Engineering, University of California, Irvine, California 92697, USA; 3 Schmid College of Science and Technology, Chapman University, Orange, California 92866, USA

## Abstract

Wearable thermoregulatory technologies have attracted widespread attention because of their potential for impacting individual physiological comfort and for reducing building energy consumption. Within this context, the study of materials and systems that can merge the advantageous characteristics of both active and passive operating modes has proven particularly attractive. Accordingly, our laboratory has drawn inspiration from the appearance-changing skin of Loliginidae (inshore squids) for the introduction of a unique class of dynamic thermoregulatory composite materials with outstanding figures of merit. Herein, we demonstrate a straightforward approach for experimentally controlling and computationally predicting the adaptive infrared properties of such bioinspired composites, thereby enabling the development and validation of robust structure–function relationships for the composites. Our findings may help unlock the potential of not only the described materials but also comparable systems for applications as varied as thermoregulatory wearables, food packaging, infrared camouflage, soft robotics, and biomedical sensing.

## INTRODUCTION

Wearable materials and systems have attracted much attention for applications as varied as fitness tracking,^1,2^ medical monitoring,^3,4^ safety and security assurance,^5,6^ communication and education,^7,8^ energy harvesting or storage,^9,10^ and personal thermal management.^11–14^ Within this context, the development of wearable thermoregulatory technologies has become a major focus in both academia and industry because of their potential for impacting individual physiological comfort and for reducing energy consumption upon widespread adoption.^13–20^ Typically, such thermoregulatory technologies have been broadly classified as passive or active based on their mode of operation.^11–14,21–24^ For instance, passive technologies are designed to statically regulate heat transfer without any energy input, often making them straightforward to implement, relatively low cost, and quite energy efficient, but they generally exhibit poor adaptability to changes in the external environment.^11–14,21,22^ In contrast, active technologies are designed to dynamically regulate heat transfer with a substantial energy input, often making them complex to implement, comparatively higher cost, and less energy efficient, but they generally feature excellent controllable responsiveness to changes in the external environment.^11–14,23,24^ As such, there exists powerful motivation for the study of wearable thermoregulatory materials and systems that can merge the advantages of both passive and active operating modes.

**FIG. 1.**
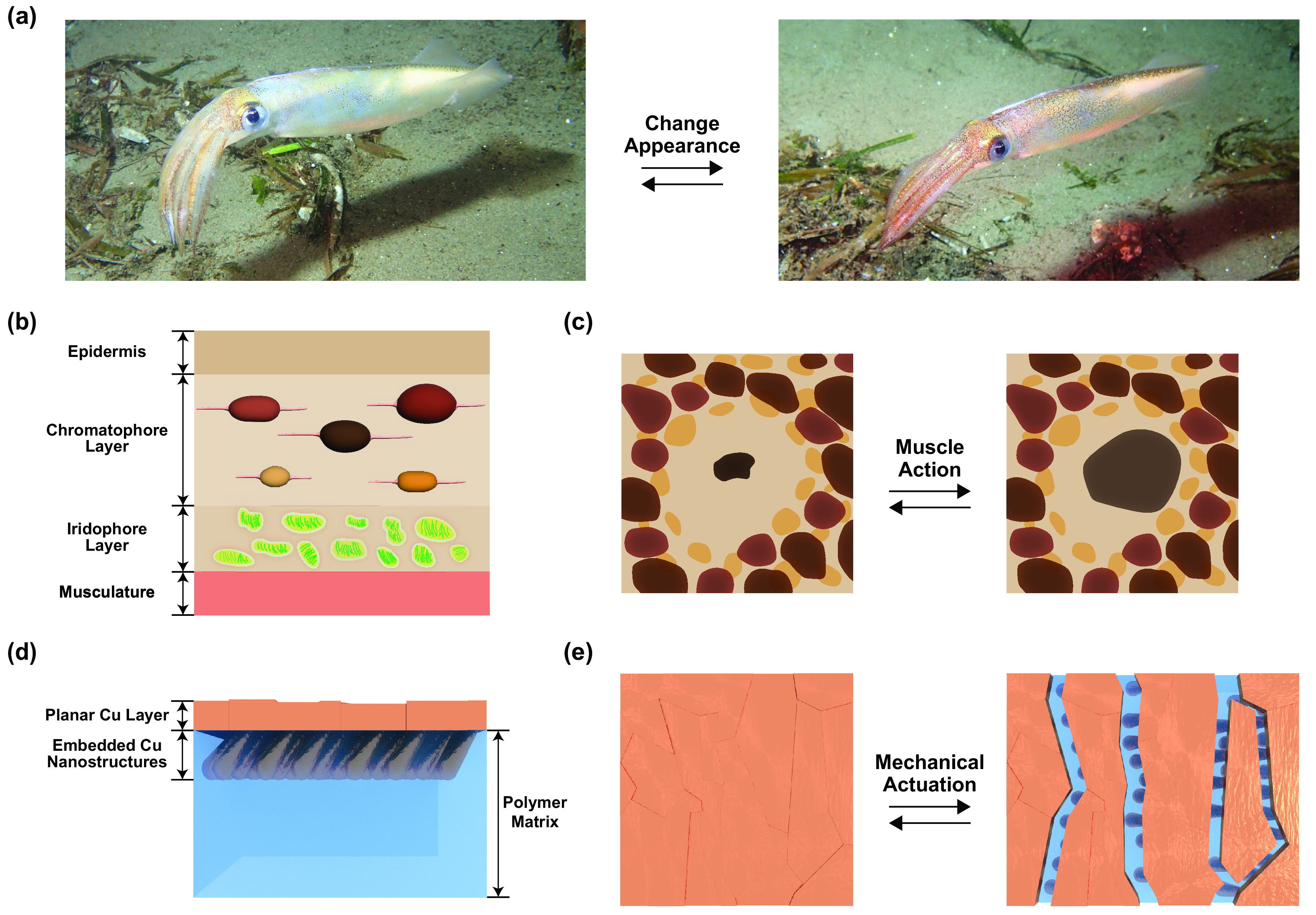
Squid skin-inspired design of the adaptive infrared composite materials. (a) Digital camera pictures of an opalescent squid changing its appearance. (b) A simplified cross-sectional illustration of the general natural architecture of squid skin, which shows the epidermis, chromatophore layer, iridophore layer, and musculature. (c) A top-view illustration of organs called chromatophores that are reversibly expanded and contracted via muscle action.^29^ (d) A cross-sectional illustration of the composite material, which shows the planar Cu layer, the embedded Cu nanostructures, and the polymer matrix. (e) A top-view illustration of the squid skin-inspired composite material for which the surface microstructure is reversibly reconfigured via mechanical actuation.^25^ Note that the pictures in (a) are reproduced with permission from S. Thiebaud, “Opalescent Inshore Squid (*Doryteuthis opalescens*),” iNaturalist, https://www.inaturalist.org/observations/65343592 (2020). Copyright 2020, Authors, licensed under a Creative Commons Attribution license.^31^

Recently, our laboratory has introduced a unique new class of thermoregulatory composite materials,^25,26^ which were engineered by drawing inspiration from the fascinating appearance-changing capabilities of Loliginidae (inshore squids) [Fig. 1(a)].^25–31^ In particular, we considered the natural architecture of squid skin [Fig. 1(b)], in which organs called chromatophores are reversibly expanded and contracted via muscle action [Fig. 1(c)],^27–29^ and we accordingly designed artificial infrared-reflecting metal–polymer composite materials [Fig. 1(d)**]**, for which the overlaid metal layer's microstructure is reversibly reconfigured via mechanical actuation [Fig. 1(e)].^25,26^ Excitingly, our bioinspired designer composite materials not only could alter their infrared transmittance by ≳20-fold but also could regulate heat fluxes by ≳30 W m^−2^ with a minimal mechanical power input.^25,26^ Additionally, when integrated into compact wearable sleeve-type devices, the materials could modulate localized body temperature changes by up to ∼10-fold as a result of actuation with applied strain.^25^ Moreover, large-area variants of such materials were scalably manufactured via standard industrial techniques at a low estimated cost of ∼US $0.1 m^−2^.^26^ However, for our composite materials, we did not previously showcase precise control over the surface microstructure, establish detailed general structure–function relationships, or develop computational methods for the prediction of their dynamic infrared properties.^25,26^

Herein, we demonstrate a straightforward approach for experimentally controlling and computationally predicting the adaptive infrared properties of our wearable bioinspired composites, thereby enabling the development and validation of robust structure-function relationships for these materials. First, we fabricate composites for which infrared-reflecting planar metal layers with variable thicknesses are overlaid on polymer matrices. Next, we evaluate our composites' strain-reconfigurable microstructural characteristics, i.e., average metal island widths and fractional metal surface coverages. In turn, we characterize our composites' strain-dependent infrared functionalities, i.e., total transmittances and reflectances. Last, we computationally simulate the composites' adaptive infrared properties. Overall, our findings may guide the continued engineering and optimization of both our composite materials and analogous systems for applications as varied as thermoregulatory wearables, food packaging, infrared camouflage, soft robotics, and biomedical sensing.

### Facile fabrication of the bioinspired composite materials

We began our efforts by fabricating composite materials consisting of nanostructured metal films embedded within an elastomeric polymer matrix, as illustrated in the supplementary material, Fig. 1. The scanning electron microscopy (SEM) images of the substrate-bound nanostructured metal films are shown in the supplementary material, Fig. 2, and the digital camera pictures and SEM images of the free-standing composite materials are shown in the supplementary material, Fig. 3. To fabricate the nanostructured metal films, we deposited infrared-reflecting planar copper (Cu) layers with variable thicknesses onto support substrates and then grew tilted columnar Cu nanostructures on top of these planar layers [supplementary material, Fig. 1(a)]. The corresponding SEM images revealed that such films consisted of planar layers with the expected thicknesses of ∼5, ∼10, ∼20, ∼50, and ∼100 nm and arrayed tilted columnar nanostructures with the anticipated heights of ∼90 nm [supplementary material, Fig. 2]. To fabricate the composite materials, we spin-coated infrared-transparent styrene–ethylene–butylene–styrene (SEBS) polymer matrices directly onto the substrate-bound nanostructured films and then delaminated the resulting architectures from the support substrates [supplementary material, Fig. 1(b)]. The corresponding digital camera pictures and SEM images revealed that such composites were globally relatively uniform and featured locally fractured topmost metal layers (supplementary material, Fig. 3). Notably, the composites' planar Cu layers could be removed via chemical treatment, confirming the presence of the embedded columnar Cu nanostructures within the polymer matrices (supplementary material, Fig. 4).^32^ Moreover, composites fabricated from planar Cu layers without nanostructures could not be reliably delaminated from the support substrate, resulting in materials with millimeter-scale defects (supplementary material, Fig. 5). Last, SEBS polymer matrices fabricated without planar Cu layers were readily delaminated from the support substrates, resulting in transparent films with no obvious large defects (supplementary material, Fig. 6). Together, our high-yield and versatile process yielded free-standing composite materials with relatively large areas of >160 cm^2^, thereby facilitating subsequent morphological and spectroscopic characterization.

### Microstructural evaluation of the bioinspired composite materials

**FIG. 2.**
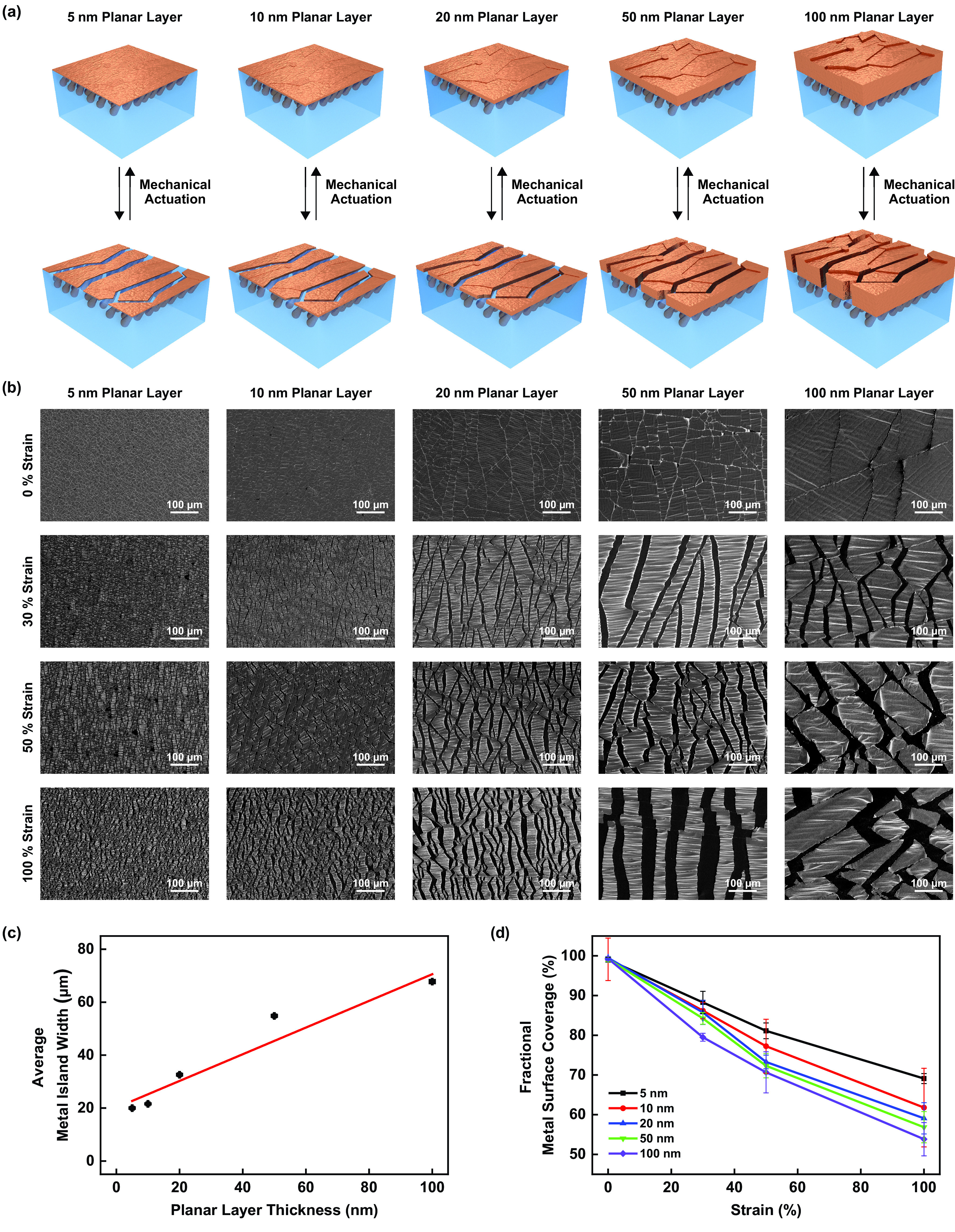
Surface microstructure of the composite materials. (a) An illustration of the composite materials with planar layer thicknesses of 5, 10, 20, 50, and 100 nm (from left to right) before (top) and after (bottom) mechanical actuation. (b) Representative top-down SEM images of the composite materials with planar layer thicknesses of 5, 10, 20, 50, and 100 nm (from left to right) under applied strains of 0%, 30%, 50%, and 100% (from top to bottom). (c) The average metal island widths for composite materials with planar layer thicknesses of 5, 10, 20, 50, and 100 nm under applied strains of 0%, 30%, 50%, and 100%. The red line corresponds to a linear fit of the data. (d) The average fractional metal surface coverages for composite materials with planar layer thicknesses of 5, 10, 20, 50, and 100 nm under applied strains of 0%, 30%, 50%, and 100%. The error bars in (c) and (d) represent the standard deviations of the mean.

After fabricating our composite materials, we qualitatively evaluated their strain-reconfigurable surface microstructures, which are illustrated in Fig. 2(a). The representative SEM images obtained for composite materials with variable planar layer thicknesses of ∼5, ∼10, ∼20, ∼50, or ∼100 nm and subjected to different uniaxial strains of 0%, 30%, 50%, or 100% are shown in Fig. 2(b). In their relaxed states (i.e., under a strain of 0%), the composites' surfaces consisted of abutted metal domains (islands) that completely covered the underlying polymer matrices, but in their actuated states (i.e., under strains of 30%, 50%, or 100%), the composites' surfaces consisted of separated metal islands that only partially covered the underlying polymer matrices [Fig. 2(b)]. Here, the composites with variable planar layer thicknesses analogously featured fractional metal surface coverages that progressively decreased with the applied strain but also did exhibit some noteworthy qualitative differences in their microstructural characteristics [Fig. 2(b)]. Specifically, the surfaces of the composites with 5 and 10 nm planar layer thicknesses were covered by small metal islands and some interspersed defects, presumably due to incomplete delamination during fabrication; the surfaces of the composites with 20 nm planar layer thicknesses were covered by intermediate-sized islands and few-to-no defects, presumably due to optimum delamination during fabrication; and the surfaces of the composites with 50 and 100 nm planar layer thicknesses were covered by large metal islands with occasional raised edges, presumably due to partial metal debonding after delamination [Fig. 2(b)]. Notably, the different types of composites all featured analogous mechanical properties (i.e., Young's moduli of ∼1–∼2 MPa and elongations to break of >900%), which were seemingly primarily dictated by the rubber-like SEBS polymer matrix and were only somewhat affected by the thicknesses of the overlaid planar Cu layers (supplementary material, Fig. 7). These experiments suggested that the morphological characteristics of our composite materials were primarily determined by a single adjustable parameter, i.e., the thickness of their planar Cu layer.

We next quantitatively evaluated the strain-reconfigurable surface microstructures of our composite materials, as illustrated in the supplementary material, Fig. 8 (see Methods for further details). The average metal island widths and average fractional metal surface coverages calculated for composite materials featuring variable planar layer thicknesses and subjected to different uniaxial strains are shown in Figs. 2(c) and 2(d), respectively. First, regardless of the applied strain, the composites' metal islands featured widths of ∼20, ∼22, ∼33, ∼55, and ∼68 *μ*m for planar layer thicknesses of 5, 10, 20, 50, and 100 nm, respectively [Fig. 2(c)]. Notably, the islands' widths increased almost linearly with the planar Cu layer thickness, in excellent agreement with classic theories developed for analyzing the fracture of thin metal films on polymers.^33,34^ Second, before actuation with strain, the composites' fractional metal surface coverages featured values of ≳94% for all planar Cu layer thicknesses, but upon actuation with strain, the composites' fractional metal surface coverages all decreased monotonically and reached values of ∼69%, ∼62%, ∼59%, ∼57%, and ∼54% (at strains of 100%) for planar layer thicknesses of 5, 10, 20, 50, and 100 nm, respectively [Fig. 2(d)]. Interestingly, the changes in the fractional metal surface coverages were directly dependent on the planar Cu layer thicknesses, in agreement with our qualitative analysis of the SEM images (vide supra). The combined analyses reinforced the notion that the morphological characteristics of our composite materials were primarily determined by a single adjustable parameter.

### Adaptive infrared functionality of the bioinspired composite materials

**FIG. 3.**
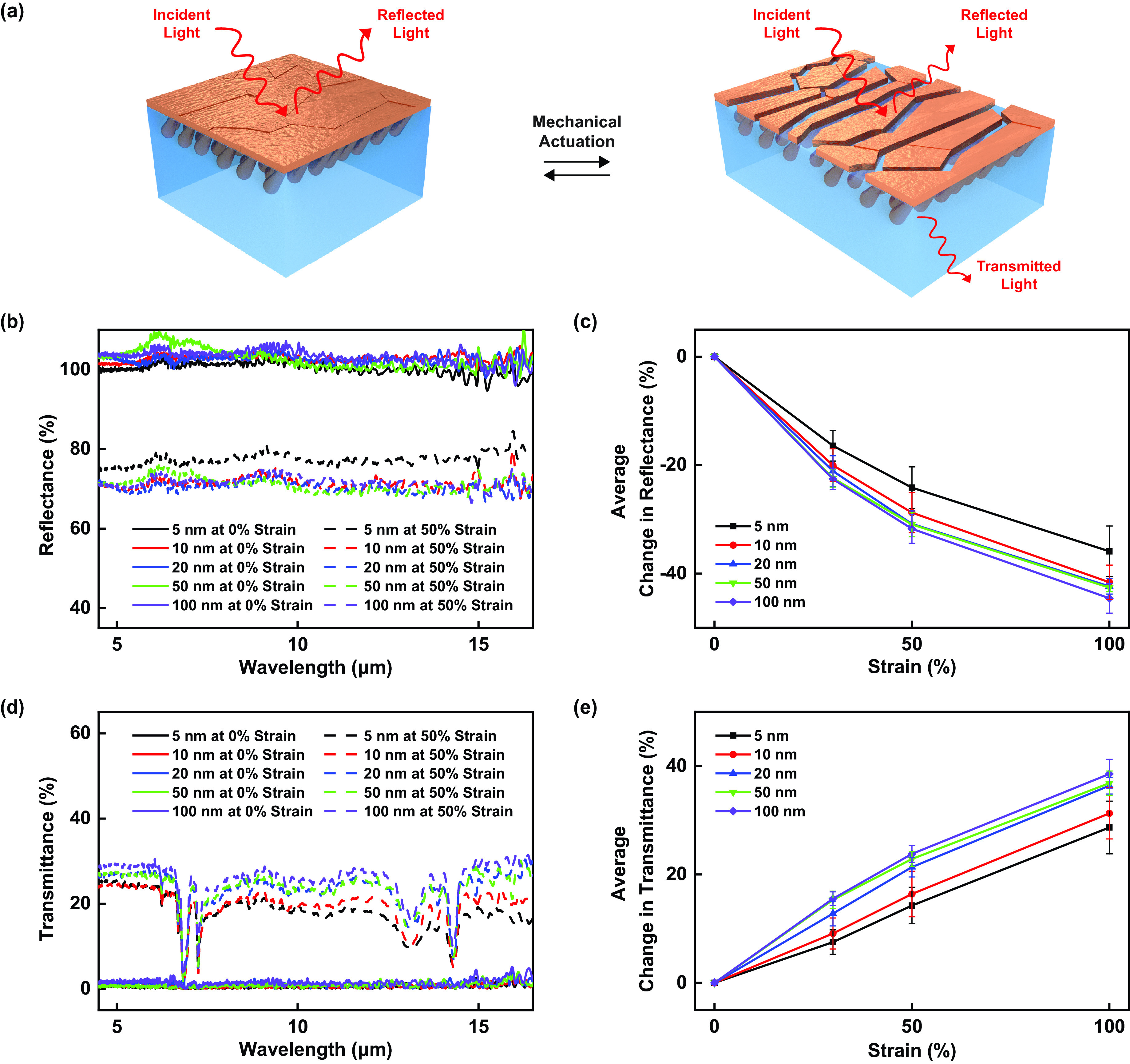
Measured adaptive infrared properties of the composite materials. (a) An illustration of the reflection and transmission of infrared light by the composite material before (left) and after (right) mechanical actuation. Note that the absorption of infrared light is not depicted for clarity. (b) The representative total infrared reflectance spectra measured for the composite materials with 5 nm (black), 10 nm (red), 20 nm (blue), 50 nm (green), and 100 nm (purple) planar layer thicknesses under applied strains of 0% (solid lines) and 50% (dashed lines). (c) The average changes in the total infrared reflectance for the composite materials with 5 nm (black), 10 nm (red), 20 nm (blue), 50 nm (green), and 100 nm (purple) planar layer thicknesses under different applied strains of ≤100%. (d) The representative total infrared transmittance spectra measured for the composite materials with 5 nm (black), 10 nm (red), 20 nm (blue), 50 nm (green), and 100 nm (purple) planar layer thicknesses under applied strains of 0% (solid lines) and 50% (dashed lines). (e) The average changes in the total infrared transmittance for the composite materials with 5 nm (black), 10 nm (red), 20 nm (blue), 50 nm (green), and 100 nm (purple) planar layer thicknesses under different applied strains of ≤100%. The error bars in (c) and (e) represent the standard deviations of the mean.

Having evaluated the surface microstructures of our composite materials, we characterized their mechanically actuated infrared-reflecting properties, which are illustrated in Fig. 3(a). The representative total infrared reflectance spectra and the average changes in the reflectance measured for unactuated and actuated composite materials with variable planar layer thicknesses are shown in Figs. 3(b) and 3(c), respectively. For composites with smaller planar layer thicknesses of 5 and 10 nm, the spectra revealed initial average total reflectances of ∼98 ± 4% and ∼101 ± 3%, respectively, at 0% strain, and decreased average total reflectances of ∼74 ± 2% and ∼73 ± 4%, respectively, at 50% strain [Fig. 3(b)]. For composites with larger planar layer thicknesses of 20, 50, and 100 nm, the spectra revealed initial average total reflectances of ∼103 ± 1%, ∼104 ± 1%, and ∼104 ± 4%, respectively, at 0% strain, and decreased average total reflectances of ∼72 ± 2%, ∼72 ± 2%, and ∼73 ± 4%, respectively, at 50% strain [Fig. 3(b)]. Here, before actuation, the spectra revealed reflectances that occasionally exceeded 100% presumably because of noise associated with environmental scattering and the use of a diffuse gold standard for calibration.^25,35–37^ More generally, the average changes in the total reflectance progressively increased as a function of the applied strain, with the most substantial reflectance modulation observed for the composites featuring the thickest planar layers [Fig. 3(c)]. Such trends presumably resulted from the relatively larger changes in the fractional metal surface coverage quantified for composites with planar layer thicknesses of ≥20 nm [Fig. 2(d)]. Notably, for composites with 5 and 10 nm planar layer thicknesses, the reflectance spectra indicated some performance degradation after repeated mechanical cycling (presumably due to the propagation of fabrication defects), and for composites with 20, 50, and 100 nm planar layer thicknesses, the reflectance spectra remained nearly unchanged after repeated mechanical cycling (presumably due to the presence of fewer defects) [supplementary material, Fig. 9(a)]. Moreover, for composites with their planar Cu layers removed and for polymer matrices without planar Cu layers, the reflectances remained relatively unchanged regardless of the applied strain, underscoring the Cu layers' critical functional roles (supplementary material, Figs. 10 and 11). Taken together, the measurements provided insight into the relationship between our composites' adaptive infrared-reflecting functionalities and reconfigurable surface microstructures.

We next spectroscopically characterized the mechanically actuated infrared-transmitting properties of our composite materials, which are illustrated in Fig. 3(a). The representative total infrared transmittance spectra and the average changes in the transmittance measured for composite materials with variable planar layer thicknesses are shown in Figs. 3(d) and 3(e), respectively. For composites with smaller planar layer thicknesses of 5 and 10 nm, the spectra revealed initial average total transmittances of ∼2 ± 1% and ∼2 ± 1%, respectively, at 0% strain, and increased average total transmittances of ∼16 ± 2% and ∼18 ± 4%, respectively, at 50% strain [Fig. 3(d)]. For composites with the larger planar layer thicknesses of 20, 50, and 100 nm, the spectra revealed initial average total transmittances of ∼1 ± 1%, ∼2 ± 1%, and ∼3 ± 2%, respectively, at 0% strain, and increased average total transmittances of ∼23 ± 2%, ∼24 ± 1%, and ∼26 ± 2%, respectively, at 50% strain [Fig. 3(d)]. Here, after actuation, the spectra revealed peaks at ∼6–∼8 *μ*m and at ∼12–∼15 *μ*m corresponding to the chemical functional groups of the partially uncovered SEBS matrices.^25,38,39^ More generally, the average changes in the total transmittance progressively increased as a function of the applied strain, with the largest modulation again observed for the composites featuring the thickest planar layers [Fig. 3(e)]. Such trends presumably resulted from the relatively larger changes in the fractional metal surface coverage quantified for the composites with planar layer thicknesses of ≥20 nm [Fig. 2(d)]. Notably, for composites with 5 and 10 nm planar layer thicknesses, the transmittance spectra indicated some performance degradation after repeated mechanical cycling (presumably due to the propagation of fabrication defects), and for composites with 20, 50, and 100 nm planar layer thicknesses, the transmittance spectra remained almost unchanged after repeated mechanical cycling (presumably due to the presence of fewer defects) [supplementary material, Fig. 9(b)]. Moreover, for composites with their planar Cu layers removed and for matrices without planar Cu layers, the transmittances remained relatively unchanged regardless of the applied strain, further reinforcing the Cu layers' critical functional roles (supplementary material, Figs. 10 and 11). Taken together, the measurements elucidated the relationship between our composites' adaptive infrared-transmitting functionalities and reconfigurable surface microstructures.

### Computational simulation of the infrared properties of the bioinspired composite materials

**FIG. 4.**
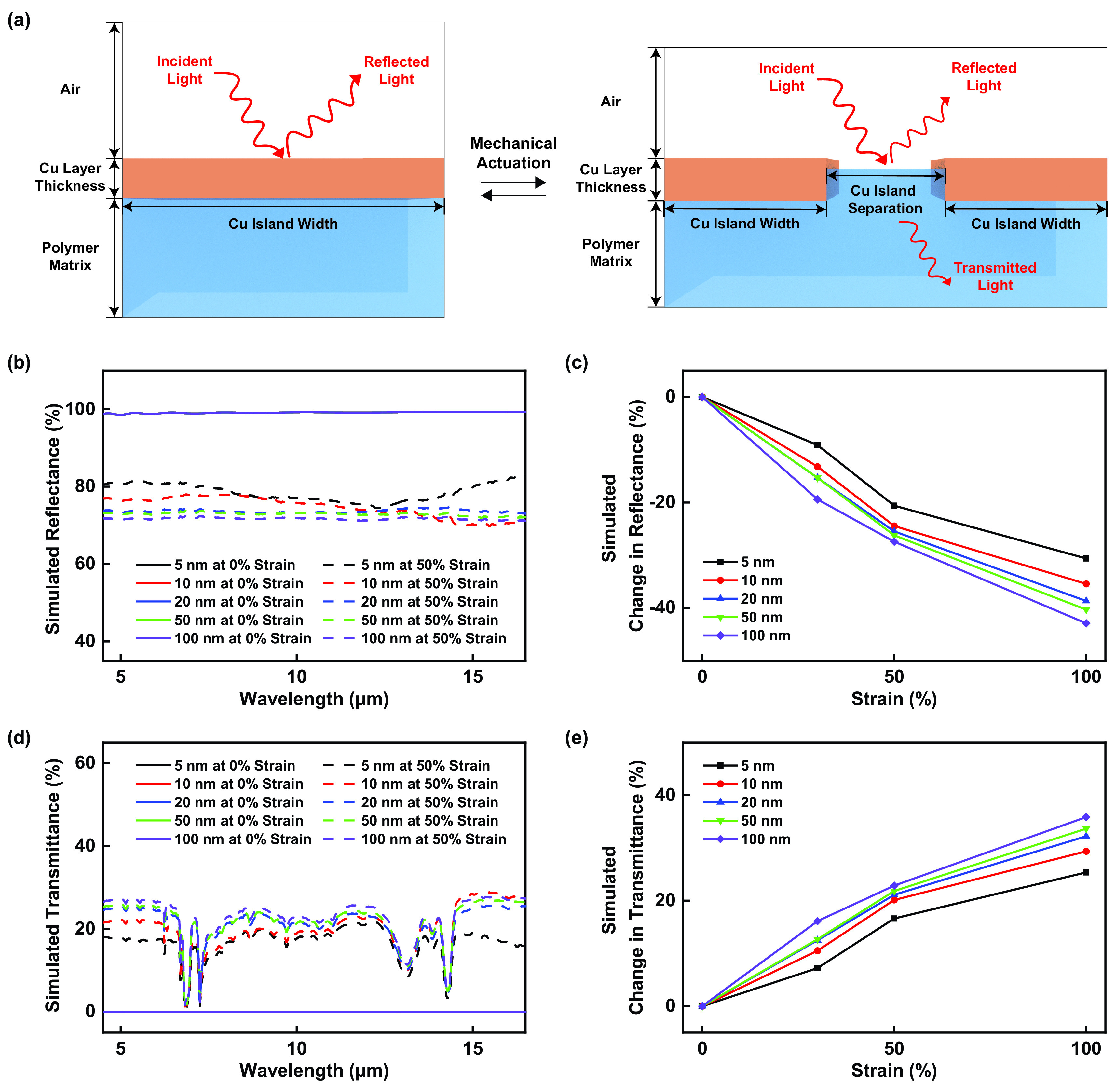
Simulated adaptive infrared properties of the composite materials. (a) An illustration of the model used to computationally simulate the reflection and transmission of infrared light by the composite material before (left) and after (right) mechanical actuation. Note that the absorption of infrared light is not depicted for clarity. (b) The simulated total infrared reflectance spectra for the composite materials with 5 nm (black), 10 nm (red), 20 nm (blue), 50 nm (green), and 100 nm (purple) planar layer thicknesses under applied strains of 0% (solid lines) and 50% (dashed lines). Note that the spectra simulated for composite materials under applied strains of 0% are overlaid on top of each other. (c) The changes in the simulated total infrared reflectance for the composite materials with 5 nm (black), 10 nm (red), 20 nm (blue), 50 nm (green), and 100 nm (purple) planar layer thicknesses under different applied strains of ≤ 100%. (d) The simulated total infrared transmittance spectra for the composite materials with 5 nm (black), 10 nm (red), 20 nm (blue), 50 nm (green), and 100 nm (purple) planar layer thicknesses for applied strains of 0% (solid lines) and 50% (dashed lines). (e) The changes in the simulated total infrared transmittance for the composite materials with 5 nm (black), 10 nm (red), 20 nm (blue), 50 nm (green), and 100 nm (purple) planar layer thicknesses under different applied strains of ≤ 100%. Note that the spectra simulated for composite materials under applied strains of 0% are overlaid on top of each other.

To better understand the adaptive infrared properties of our composite materials, we computationally simulated their strain-dependent infrared reflectance spectra via a straightforward model, as illustrated in Fig. 4(a) (see Methods for further details). The calculated total infrared reflectance spectra and the calculated changes in the reflectance obtained for the various composites are shown in Figs. 4(b) and 4(c), respectively. For composites with smaller planar layer thicknesses of 5 and 10 nm, the simulated spectra revealed average total reflectances of ∼99% and ∼99%, respectively, at 0% strain, with the reflectances decreasing to values of ∼79% and ∼75%, respectively, at 50% strain [Fig. 4(b)]. For composites with larger planar layer thicknesses of 20, 50, and 100 nm, the simulated spectra revealed average total reflectances of ∼99%, ∼99%, and ∼99%, respectively, at 0% strain, with the reflectances decreasing to values of ∼74%, ∼73%, and ∼72%, respectively, at 50% strain [Fig. 4(b)]. In general, the calculated changes in the total reflectance progressively increased with the applied strain and were maximized for the composites featuring the thickest planar layers [Fig. 4(c)]. Notably, the simulated total reflectance spectra and calculated total reflectance modulation trends were in close agreement with our experimental measurements [supplementary material, Fig. 12(a)]. The computational simulations thus provided powerful validation for our experimental observations and confirmed the fact that our composites' reconfigurable surface microstructure governed their adaptive infrared-reflecting functionalities.

We next computationally simulated the strain-dependent infrared transmittance spectra via the same model, as illustrated in Fig. 4(a) (see Methods for further details). The calculated total infrared transmittance spectra and the calculated changes in the transmittance for the various composites are shown in Figs. 4(d) and 4(e), respectively. For composites with the smaller planar layer thicknesses of 5 and 10 nm, the simulated spectra revealed average total transmittances of ∼0% and ∼0%, respectively, at 0% strain, with the transmittances increasing to values of ∼17% and ∼20%, respectively, at 50% strain [Fig. 4(d)]. For composites with larger planar layer thicknesses of 20, 50, and 100 nm, the simulated spectra revealed average total transmittances of ∼0%, ∼0%, and ∼0%, respectively, at 0% strain, with the transmittances increasing to values of ∼21%, ∼22%, and ∼23%, respectively, at 50% strain [Fig. 4(d)]. In general, the calculated changes in the total transmittance progressively increased with the applied strain and were maximized for the composites featuring the thickest planar layers [Fig. 4(e)]. Notably, the simulated total transmittance spectra and calculated total transmittance modulation trends were in close agreement with our experimental measurements [supplementary material, Fig. 12(b)]. The computational simulations again provided powerful validation for our experimental observations and further reinforced the fact that our composites' reconfigurable surface microstructure governed their adaptive infrared-transmitting functionalities.

## DISCUSSION AND CONCLUSION

In summary, we have validated a straightforward methodology for experimentally controlling and computationally predicting the adaptive infrared properties of our squid skin-inspired wearable composite materials, and our findings hold significance for multiple reasons. Specifically, we have demonstrated that the surface microstructure of our composite materials can be controlled via modification of a single parameter (i.e., the planar layer thickness) during fabrication. This discovery establishes the planar layer as a critical consideration during the high-throughput scalable manufacturing of our composites. Additionally, we have shown a direct relationship between the reconfigurable surface microstructure of our composite materials and their tunable infrared-reflecting and infrared-transmitting properties. This observation provides additional nuanced fundamental insight into the origins of our composites' adaptive infrared functionalities. Moreover, we have developed a straightforward computational model that precisely predicts the infrared properties of our composite materials. This advance will enable the targeted design of improved variants of our composites from arbitrary combinations of metals and polymers. Importantly, our key reported outcomes with respect to fabrication, structure–function relationships, and computational modeling should prove valuable for the engineering and optimization of other adaptive infrared platforms. As such, the described findings may help unlock the potential of not only our composite materials but also comparable systems for applications as varied as thermoregulatory wearables, food packaging, infrared camouflage, soft robotics, and biomedical sensing.

## METHODS

### Fabrication of the nanostructured films

The nanostructured Cu films were fabricated according to reported protocols.^25,26^ First, a planar Cu layer (Kurt J. Lesker) with a thickness of 5, 10, 20, 50, or 100 nm was electron beam evaporated onto a 6-in. diameter silicon wafer (University Wafer) at a deposition angle of α = 0° by using an Angstrom Engineering EvoVac system (Angstrom Engineering). Next, ∼90-nm tilted columnar Cu nanostructures were electron beam evaporated onto the planar Cu layer at a deposition angle of α = 89°. The analogous planar Cu layers without nanostructures were fabricated by omitting the nanostructure formation step. The resulting nanostructured films were characterized with SEM imaging and used for the fabrication of the composite materials.

### Fabrication of the composite materials

The composite materials were fabricated according to reported protocols.^25,26^ First, a 50% (w/w) solution of styrene–ethylene–butylene–styrene (SEBS) block copolymer (G1645, Kraton LLC) in toluene (Fisher Chemical) was spin-coated onto the silicon wafer-bound nanostructured Cu films. Next, the resulting nanostructured composites were heat-treated at 60 °C for 10 min. to remove any residual solvent. Last, the nanostructured composites with typical thicknesses of ∼30 *μ*m were manually delaminated (detached) from the substrate as free-standing materials by means of an attached plastic frame. The composites lacking planar Cu layers were fabricated by chemically etching free-standing nanostructured composites with a 0.2% FeCl_3_ solution (Fisher Scientific).^32^ The composites lacking Cu nanostructures were fabricated from planar Cu layers on silicon wafers. The SEBS polymer matrices (films) were fabricated by spin-coating SEBS solutions directly onto clean silicon wafers. The composite materials obtained via these procedures were characterized with digital camera imaging, SEM imaging, tensile testing, infrared spectroscopy characterization, and stability testing.

### Digital camera imaging of the composite materials

The visible appearances of the free-standing composite materials were characterized with digital camera imaging. The pictures were obtained with either a built-in phone camera (iPhone, Apple) or a digital single-lens reflex camera (PowerShot SX520, Canon) and were analyzed with the Photoshop (Adobe) software package. The digital camera pictures were collected routinely and enabled detailed global inspection of the composite materials.

### SEM imaging of the nanostructured films and composite materials

The morphologies of the nanostructured films and free-standing composite materials were characterized with SEM. The images were obtained with a Magellan 400 XHR SEM (FEI) and were analyzed with the Photoshop (Adobe) software package. For imaging of the nanostructured films, the pristine samples (typical sizes of 2 × 6 mm^2^) were not modified. For imaging of the composite materials, the samples (typical sizes of 2 × 6 mm^2^) were subjected to the appropriate strain (0%, 30%, 50%, or 100%), fixed with an epoxy resin (Ted Pella), and sputter-coated with a ∼3-nm film of iridium. The nanostructured films and composite materials were typically imaged at accelerating voltages of 10 and 5 kV, respectively, at various magnifications. The SEM images were collected routinely and enabled quality control, thickness measurements, and surface microstructure analyses.

### Computational analysis of the surface microstructure of the composite materials

The surface microstructures of the composite materials were analyzed by using standard image processing methods. The SEM images obtained for the composite materials were segmented/binarized into distinct metal and polymer regions via two approaches.^40^ For the composites at 0% strain, the SEM images were segmented in ImageJ (NIH) by a single-step process of applying a threshold to separate the material into two different types of regions corresponding to the metal islands and polymer matrices. For the composites at 30%, 50%, and 100% strain, the SEM images were segmented in Mathematica (Wolfram Research) via a multistep process, as schematically illustrated in the supplementary material, Fig. 8. First, the ridgelines in the images corresponding to the second derivative maxima in the y-direction were smoothed/blurred via a localized filter in order to attenuate any brightness/contrast distortion, and, in tandem, the island edges in the images corresponding to the second order derivative maxima in the x-direction were sharpened in order to enhance the desired boundaries. Second, the processed images’ brightnesses were enhanced to improve the contrast between the different identified microstructural features. Third, the brightened images were further smoothed with edge preservation. Fourth, the smoothed images were segmented (binarized) into the two different types of regions corresponding to the metal islands and polymer matrices by using the native Mathematica clustering algorithm.

The average metal island widths on the composites' surfaces $W_{Cu}$ were calculated from the SEM images by using the following equations:

$$W_{Cu}=W_{\text{pixel}}\times \frac{N_{\text{green}}}{N},$$ (1)

where $W_{\text{pixel}}$ is the width of a pixel, $N_{\text{green}}$ is the total number of green pixels (corresponding to the metal islands), and N is the total number of green pixel lines that span the metal islands in the horizontal direction.

The fractional metal surface coverages ρ, i.e., the ratio of the metal islands' areas to the entire surfaces' areas, were calculated from the processed SEM images by using the following equation:

$$\rho =\frac{N_{\text{green}}}{N_{\text{green}}+N_{\text{gray}}}\times 100\%,$$ (2)

where $N_{\mathrm{green}}$ is the total number of green pixels (corresponding to all overlaid metal islands) and $N_{\text{gray}}$ is the total number of gray pixels (corresponding to the exposed polymer matrices). The calculations enabled a quantitative evaluation of the composite materials' surface microstructures.

### Tensile testing of the composite materials

The mechanical properties of the composite materials were characterized according to standard protocols.^25,26,41^ The experiments were performed by using a 3365 Universal Testing System (Instron) and were analyzed with Origin 8.5 (OriginLab) software packages. The composites were mounted in the grips of the instrument, subsequently subjected to three cycles of 0–100% uniaxial strain at an elongation rate of 15 mm s^−1^ and then stretched from 0% strain to their breaking (yield) strain at a rate of 30 mm s^−1^. The Young's moduli were calculated from the linear regions of the engineering stress vs strain curves at strain values of 30%. The tensile testing measurements were performed on at least three different composite materials of each type, with similar results obtained in each instance.

### Infrared spectroscopy of the composite materials

The infrared properties of the composite materials were characterized according to standard protocols.^25,26,42^ The total infrared reflectance and transmittance spectra were obtained by using a Frontier Fourier transform infrared (FTIR) spectrometer (PerkinElmer) outfitted with a mid-infrared integrating sphere (Pike Technologies) and were analyzed with the Spectrum (PerkinElmer) and Origin 8.5 (OriginLab) software packages. The measurements were referenced to a National Institute of Standards and Technology (NIST)-verified Pike Technologies Diffuse Gold Standard. The composite materials were mounted on home-built size-adjustable stages, which allowed for the application of strains between 0% and 100%. The total reflectance spectra were collected at an illumination angle of 12°, and the total transmittance spectra were collected at a normal illumination angle. The spectra were collected in an indoor environment at room temperature, i.e., typically ∼20 °C. The average reflectance and transmittance value changes were calculated for the composites over the wavelength range of 4.5–16.5 *μ*m by subtracting the average values at strains of 0% from the average values at strains of >0%. The experiments were performed on at least eight different composite materials of each type, with similar results obtained in each instance.

### Stability testing of the composite materials

The functional stabilities of the composite materials were characterized with a combination of infrared spectroscopy and mechanical cycling according to standard protocols.^25,26^ First, the composites' total reflectance and transmittance spectra were measured at uniaxial strains of 0% and 50% with a Frontier FTIR spectrometer (PerkinElmer) outfitted with a Pike Technologies Mid-Infrared Integrating Sphere, as described above. Next, the composites were cycled 1000 times between applied uniaxial strains of 0% and 50% at a frequency of 1 Hz using an ESM303 tension/compression test stand (MARK-10). In turn, the composites' total reflectance and transmittance spectra were measured again at uniaxial strains of 0% and 50%. Last, the spectra were comparatively analyzed with the Spectrum (PerkinElmer) and Origin 8.5 (OriginLab) software packages. The stability testing measurements were performed on at least three composite materials of each type, with similar results obtained in each instance.

### Computational modeling of the adaptive infrared properties of the composite materials

The infrared properties of the composite materials were computationally modeled by using the Electromagnetic Waves, Frequency Domain Interface of the Wave Optics Module in the COMSOL Multiphysics 5.6 (COMSOL) software package.^43^ The reflectances and transmittances of the various Cu- and SEBS-based composite materials before and after actuation were calculated by considering the reflection and transmission of infrared light in air from a two-dimensional unit cell consisting of uniformly distributed and periodic surface microstructures, as schematically illustrated in Fig. 4(a). The simulations and calculations required (1) the thickness of the Cu islands $H_{Cu}$, (2) the average separation between the Cu islands $W_{\text{sep}}$, (3) the complex refractive index of air ${\bar{n}}_{\text{air}}$, (4) the complex refractive index of Cu ${\bar{n}}_{Cu}$, and (5) the complex refractive index of SEBS ${\bar{n}}_{\mathrm{SEBS}}$.

First, the thickness of the Cu islands $H_{Cu}$ was estimated by using the following equation:

$$H_{\mathrm{Cu}}=H_{\text{planar}}+H_{\text{nano}},$$ (3)

where $H_{\text{planar}}$ is the thickness of the SEBS-overlaid planar Cu layer extracted from the SEM images, and $H_{\text{nano}}$ is the height of the slanted SEBS-embedded Cu nanostructures extracted from the SEM images.

Second, the average separation between the Cu islands $W_{\text{sep}}$ was estimated by using the following equation:

$$W_{\text{sep}}=W_{\mathrm{Cu}}\times \frac{1-\rho }{\rho },$$ (4)

where $W_{Cu}$ is the average Cu island width extracted from the SEM images, and ρ is the fractional metal surface coverage extracted from the SEM images.

Third, the complex refractive index of air ${\bar{n}}_{\text{air}}$ was estimated by using the following equation:

$${\bar{n}}_{\text{air}}=n_{\text{air}}+ik_{\text{air}},$$ (5)

where $n_{\text{air}}$ is the real part of the complex refractive index of air reported in the literature, and $k_{\text{air}}$ is the imaginary part of the complex refractive index of air reported in the literature.^44^

Fourth, the complex refractive index of Cu ${\bar{n}}_{\mathrm{Cu}}$ was estimated by using the following equation:

$${\bar{n}}_{\mathrm{Cu}}=n_{\mathrm{Cu}}+ik_{\mathrm{Cu}},$$ (6)

where $n_{\mathrm{Cu}}$ is the real part of the complex refractive index of Cu reported in the literature, and $k_{\mathrm{Cu}}$ is the imaginary part of the complex refractive index of Cu reported in the literature.^45^

Fifth, the complex refractive index of SEBS ${\bar{n}}_{\mathrm{SEBS}}$ was estimated by using the following equation:

$${\bar{n}}_{\mathrm{SEBS}}=n_{\mathrm{SEBS}}+ik_{\mathrm{SEBS}},$$ (7)

where $n_{\mathrm{SEBS}}$ is the real part of the complex refractive index of SEBS calculated by using Eqs. (8), (9), and (10), and $k_{\mathrm{SEBS}}$ is the imaginary part of the complex refractive index of SEBS calculated by using Eqs. (9) and (10).^46^

The real part $n_{\mathrm{SEBS}}$ of the complex refractive index of SEBS was estimated by using the following equation:

$$n_{\mathrm{SEBS}}=\frac{1+r}{1-r}+\sqrt{{\left(\frac{1+r}{1-r}\right)}^{2}-1-{k_{SEBS}}^{2}},$$ (8)

where r is the reflectance at the air–SEBS interface calculated by using Eq. (9).^46^

The reflectance at the air–SEBS interface r was estimated by using the following equation:

$$r=\frac{2+{T_{\mathrm{SEBS}}}^{2}-{\left(1-R_{\mathrm{SEBS}}\right)}^{2}-\sqrt{{\left(2+{T_{\mathrm{SEBS}}}^{2}-{\left(1-R_{\mathrm{SEBS}}\right)}^{2}\right)}^{2}-4R_{\mathrm{SEBS}}\left(2-R_{\mathrm{SEBS}}\right)}}{2\left(2-R_{\mathrm{SEBS}}\right)}.$$ (9)

where $T_{\mathrm{SEBS}}$ is the measured total transmittance of SEBS, and $R_{\mathrm{SEBS}}$ is the measured total reflectance of SEBS.^46^

The imaginary part $k_{\mathrm{SEBS}}$ of the complex refractive index of SEBS was estimated by using the following equation:

$$k_{\mathrm{SEBS}}=\frac{\lambda }{4\pi h}\ln(\frac{rT_{\mathrm{SEBS}}}{R_{\mathrm{SEBS}}-r}),$$ (10)

where λ is the wavelength of the incident light, and h is the thickness of the SEBS layer.^46^

The reflectance and transmittance value changes were calculated for the various composites over the wavelength range of 4.5–16.5 *μ*m by subtracting the average values at strains of 0% from the average values at strains of >0%. The combined calculations enabled a quantitative evaluation of the composite materials' adaptive infrared properties.

## SUPPLEMENTARY MATERIAL

See the supplementary material for supplementary Figs. 1–12, which show additional details of the fabrication, digital camera imaging, SEM imaging, tensile testing, computational analysis, stability testing, infrared spectroscopy characterization, and computational simulations for the described composite materials.

## Data Availability

The data that support the findings of this study are available within the article and its supplementary material.
